# Elucidating the Impact of Deleterious Mutations on IGHG1 and Their Association with Huntington’s Disease

**DOI:** 10.3390/jpm14040380

**Published:** 2024-04-01

**Authors:** Alaa Shafie, Amal Adnan Ashour, Farah Anjum, Anas Shamsi, Md. Imtaiyaz Hassan

**Affiliations:** 1 Department of Clinical Laboratory Sciences, College of Applied Medical Sciences, Taif University, P.O. Box 11099, Taif 21944, Saudi Arabia; a.shafie@tu.edu.sa (A.S.); farahanjum@tu.edu.sa (F.A.); 2 Department of Oral and Maxillofacial Surgery and Diagnostic Sciences, Faculty of Dentistry, Taif University, P.O. Box 11099, Taif 21944, Saudi Arabia; a.a.ashour@tu.edu.sa; 3 Center of Medical and Bio-Allied Health Sciences Research (CMBHSR), Ajman University, Ajman P.O. Box 346, United Arab Emirates; 4 Centre for Interdisciplinary Research in Basic Sciences, Jamia Millia Islamia, Jamia Nagar, New Delhi 110025, India

**Keywords:** Huntington’s disease, immunoglobulin heavy constant gamma 1, deleterious mutations, amino acid substitutions, bioinformatics analysis

## Abstract

Huntington’s disease (HD) is a chronic, inherited neurodegenerative condition marked by chorea, dementia, and changes in personality. The primary cause of HD is a mutation characterized by the expansion of a triplet repeat (CAG) within the *huntingtin* gene located on chromosome 4. Despite substantial progress in elucidating the molecular and cellular mechanisms of HD, an effective treatment for this disorder is not available so far. In recent years, researchers have been interested in studying cerebrospinal fluid (CSF) as a source of biomarkers that could aid in the diagnosis and therapeutic development of this disorder. Immunoglobulin heavy constant gamma 1 (IGHG1) is one of the CSF proteins found to increase significantly in HD. Considering this, it is reasonable to study the potential involvement of deleterious mutations in *IGHG1* in the pathogenesis of this disorder. In this study, we explored the potential impact of deleterious mutations on *IGHG1* and their subsequent association with HD. We evaluated 126 single-point amino acid substitutions for their impact on the structure and functionality of the IGHG1 protein while exploiting multiple computational resources such as SIFT, PolyPhen-2, FATHMM, SNPs&Go mCSM, DynaMut2, MAESTROweb, PremPS, MutPred2, and PhD-SNP. The sequence- and structure-based tools highlighted 10 amino acid substitutions that were deleterious and destabilizing. Subsequently, out of these 10 mutations, eight variants (Y32C, Y32D, P34S, V39E, C83R, C83Y, V85M, and H87Q) were identified as pathogenic by disease phenotype predictors. Finally, two pathogenic variants (Y32C and P34S) were found to reduce the solubility of the protein, suggesting their propensity to form protein aggregates. These variants also exhibited higher residual frustration within the protein structure. Considering these findings, the study hypothesized that the identified variants of IGHG1 may compromise its function and potentially contribute to HD pathogenesis.

## 1. Introduction

Huntington’s disease (HD) presents an enduring challenge within the landscape of neurodegenerative disorders [1]. It is a hereditary disorder characterized by a relentless progression of motor dysfunction, cognitive deterioration, and psychiatric disturbances [2]. With an incidence of approximately 5 to 10 cases per 100,000 individuals worldwide, HD represents one of the most prevalent autosomal dominant inherited neurodegenerative conditions [3]. The disease compromises language abilities, and there is evidence that the pragmatic aspects of communication are impaired in the early stages of the disease [4]. The disease progression follows a characteristic pattern of brain atrophy, beginning in the basal ganglia structures [4]. HD is devastating to patients and their families, with autosomal dominant inheritance, onset (typically) in the prime of adult life, a progressive course, and a combination of motor, cognitive, and behavioral features [5]. Motor dysfunction normally manifests as involuntary choreiform movements, which may progress to dystonia, bradykinesia, and akinetic-rigid symptoms in the advanced stages of the disease [6]. Cognitive decline in HD encompasses deficits in executive function, attention, visuospatial processing, and working memory, culminating in profound dementia as the disease advances [7]. Additionally, behavioral features, such as depression, anxiety, irritability, and apathy, are prevalent throughout the disease [8].

Overall, HD remains a challenging condition with limited therapeutic options [9]. At the neuropathological level, HD is characterized by the selective degeneration of neurons within the striatum, particularly the medium spiny neurons of the caudate nucleus and putamen [10]. This neurodegeneration is accompanied by widespread atrophy of the cerebral cortex, thalamus, and other subcortical structures, leading to global alterations in brain structure and function. The underlying molecular mechanisms driving neuronal dysfunction and death in HD involve a complex interplay of genetic, epigenetic, and environmental factors [11]. An expansion of CAG trinucleotide causes HD repeats within the *huntingtin* gene located on chromosome 4p16.3, leading to an elongated polyglutamine tract in the huntingtin protein [12]. The length of the CAG repeat tract inversely correlates with the age of disease onset, with longer repeats typically associated with earlier onset and more severe clinical phenotypes. While the expanded CAG repeat is the primary genetic determinant of HD, numerous modifier genes and environmental factors influence disease penetrance, expressivity, and progression [13].

Despite decades of dedicated research, the precise mechanisms governing HD pathogenesis remain incompletely elucidated [14]. There is an urgent need for disease-modifying therapies that can halt or slow the progression of HD, making elucidating disease mechanisms and identifying therapeutic targets a top priority in HD research. In recent years, advances in genomic technologies, neuroimaging techniques, and biomarker discovery have provided unprecedented insights into the pathobiology of HD [15]. Genome-wide association studies (GWASs) have identified genetic variants associated with HD onset, progression, and phenotypic variability [16,17]. Cerebrospinal fluid (CSF) has emerged as a promising arena for biomarker discovery that offers glimpses into the molecular complexities of various neurodegenerative diseases, including HD [18]. CSF serves as a reservoir of proteins, metabolites, and other molecules that mirror the pathophysiological alterations occurring within the central nervous system (CNS) [19]. Among the CSF proteins, immunoglobulin heavy constant gamma 1 (IGHG1) has been implicated in the disease process due to its elevated levels in HD [20,21,22].

Recent studies have shifted towards the genetic foundations of HD that explore the potential influence of deleterious mutations in driving disease pathogenesis [23,24]. The *IGHG1* gene that encodes the gamma-1 chain of the immunoglobulin G (IgG) has emerged as a potential target, with emerging evidence suggesting its involvement in HD etiology [21,22]. Understanding the effects of amino acid substitutions within a protein is vital for elucidating their contributions to disease pathogenesis and identifying potential therapeutic targets [25,26,27].

In this study, we analyzed deleterious mutations within IGHG1 and their association with HD pathogenesis in detail. We explored amino acid substitutions’ structural and functional consequences on the IGHG1 protein while exploiting advanced computational tools and bioinformatics resources. Through an extensive examination of 126 single-point variations, we aimed to identify “high-confidence variants” in *IGHG1* with significant implications for HD pathophysiology.

Our approach encompasses a multifaceted analysis that integrates predictions from multiple computational platforms such as SIFT [28], PolyPhen-2 [29], FATHMM [30], SNPs&Go [31], mCSM [32], DynaMut2 [33], MAESTROweb [34], PremPS [35], MutPred2 [36], and PhD-SNP [37]. By studying the impact of these variants on protein structure, stability, and function, we seek to delineate their potential role in the disrupting IGHG1-mediated processes implicated in HD pathogenesis. We focus on variants localized within specific domains of the IGHG1 protein, particularly the Ig-like 1 domain, which plays a crucial role in protein-protein interactions and immune function. By pinpointing mutations with a propensity to impair protein solubility and disrupt essential protein dynamics, we aim to elucidate their contribution to HD pathology. Ultimately, our findings promise to uncover novel insights into the molecular mechanisms driving HD. By elucidating the role of deleterious variants in *IGHG1* and their association with HD, we aim to lay the foundation for developing targeted therapies and precision medicine approaches to mitigate the devastating impact of this debilitating disorder.

## 2. Materials and Methods

### 2.1. Data Retrieval and Web Resources

The protein sequence corresponding to human IGHG1 was sourced from the UniProt protein database (Accession ID: P01857) [38]. A comprehensive compilation of individual amino acid substitutions within IGHG1 was curated by accessing data from dbSNP [39], Ensembl [40], and the relevant PubMed literature [41] (Supplementary Data). To ensure data integrity, duplicates were carefully removed from the variant dataset. The three-dimensional structure of human IGHG1, specifically its actin-binding domain, was retrieved from the RCSB Protein Data Bank (PDB ID: 4LLD). Various computational tools were employed to conduct diverse predictions and calculations, with detailed discussions provided in the subsequent sections.

### 2.2. Sequence-Based Predictions

#### 2.2.1. PolyPhen2

PolyPhen-2 (Polymorphism Phenotyping v2) is a bioinformatics tool that forecasts the potential effects of amino acid substitutions on protein structure and functionality [29]. By utilizing this resource, we assessed the impact of amino acid substitutions within the IGHG1 protein sequence. It evaluates these substitutions by integrating both the comparative and physical attributes of amino acids and estimates the likelihood of these substitutions disrupting the native conformation of the protein. Utilizing a combination of sequence, structural, and evolutionary data, PolyPhen-2 employs machine learning algorithms trained on a vast dataset of known polymorphisms to categorize variants into distinct categories based on their predicted impact, such as “benign”, “possibly damaging”, or “probably damaging”.

#### 2.2.2. SIFT

SIFT (sorting intolerant from tolerant) is a bioinformatics tool utilized in computational biology and genetics to predict the potential impact of amino acid substitutions on protein function [28]. Our study employed SIFT to prioritize genetic variants within the IGHG1 protein sequence. Like PolyPhen-2, SIFT aids in prioritizing genetic variants by classifying them as either tolerated or intolerant based on their effect on protein structure and function. It achieves this by comparing the amino acid at the position of interest with those found in related proteins across diverse species. SIFT assigns a score to each amino acid substitution, with lower scores indicating a higher likelihood of being deleterious. Variants are classified as intolerable if the SIFT score is 0.05 or lower.

#### 2.2.3. FATHMM

FATHMM (functional analysis through hidden Markov models) is a bioinformatics tool for predicting the functional impacts of genetic variants, particularly those occurring in the coding regions of the genome [30]. In our study, we harnessed FATHMM to assess the potential impact of amino acid substitutions within the IGHG1 protein. By employing a hidden Markov model (HMM) approach, FATHMM assesses the potential impact of variants on protein structure and function. It evaluates variants based on diverse features, encompassing evolutionary conservation, the physicochemical properties of amino acids, and genomic context. By amalgamating these features into its model, FATHMM predicts the potential functional impact of a given variant. The output of FATHMM typically includes a score or prediction, indicating whether a genetic variant is likely to be deleterious or tolerated. A high FATHMM score suggests that the variant is likely to be tolerated. In contrast, a low score indicates that the variant may have functional consequences and could be associated with diseases.

#### 2.2.4. SNPs&GO

SNPs&GO is a web server that employs a support vector machine (SVM) for identifying deleterious single-point amino acid substitutions [31]. Here, we utilized SNPs&GO to predict the impact of amino acid substitutions within the IGHG1 protein sequence. The SVM classifier amalgamates protein sequence, profile, and functional data to discriminate between disease-associated and neutral variants using gene ontology (GO) annotations. SNPs&GO harnesses machine learning algorithms trained on various features derived from protein sequence and structure to predict the impact of SNPs. These features encompass amino acid physicochemical properties, evolutionary conservation, protein domain information, and structural annotations. An SNPs&GO score exceeding 0.5 signifies a substitution likely to induce disease. Additionally, the tool yields output from supplementary resources such as PANTHER and PhD-SNP.

### 2.3. Structure-Based Predictions

#### 2.3.1. mCSM

mCSM (mutation cutoff scanning matrix) is a web tool utilized in structural bioinformatics to predict the effects of variants on protein stability [32]. It operates through a graph-based method to evaluate single-point amino acid substitutions. The predictive models of mCSM are developed by leveraging environmental data gleaned from the atomic distance patterns of diverse residues. This tool enhances our comprehension of disease-associated variants spanning a spectrum of proteins. A mCSM score (ΔΔ*G*) below 0 indicates a mutation that profoundly influences protein structure.

#### 2.3.2. MAESTROweb

MAESTROweb, available at https://pbwww.services.came.sbg.ac.at/maestro/web (accessed on 15 January 2024), is an online platform facilitating protein structure-based prediction of mutation effects [34]. It is used to discern how variants impact protein stability. MAESTROweb predicts the functional ramifications of genetic variations, especially concerning human diseases. It amalgamates several computational tools to scrutinize the impact of variants on protein stability. By employing diverse computational algorithms and methods, including machine learning techniques and structural bioinformatics approaches, MAESTROweb provides insights into the functional consequences of variants. A score below 0 denotes an anticipated alteration in the stability of protein due to the amino acid substitutions.

#### 2.3.3. PremPS

PremPS ((https://lilab.jysw.suda.edu.cn/research/PremPS) accessed on 15 January 2024), also evaluates the impact of amino acid substitutions in proteins utilizing multiple approaches such as multiple sequence alignment (MSA), protein structure, and a deep learning model [35]. It offers predictions concerning the potential impact of genetic variants on protein function and disease, specifically focusing on whether a single amino acid substitution is likely to be harmful or tolerated based on the sequence information of the protein. Users input the amino acid sequence of the protein, specify the position and the amino acid substitution, and PremPS calculates a prediction score, indicating the likelihood of the substitution being deleterious or tolerated.

#### 2.3.4. DynaMut2

DynaMut2 (http://biosig.unimelb.edu.au/dynamut2, accessed on 15 January 2024) is another predictive tool tailored for estimating protein stability [33]. By exploiting the data sourced from the ProTherm database, DynaMut2 furnishes predictions for both single and multiple amino acid substitutions. Our analysis primarily focuses on its single mutation prediction capability. DynaMut2 provides users with easily interpretable results, encompassing predictions of the impact of amino acid substitution on stability and dynamic properties, along with visualizations that aid understanding of the structural context of the variants.

### 2.4. Pathogenicity Prediction

#### 2.4.1. PhD-SNP

PhD-SNP (https://snps.biofold.org/phd-snp/phd-snp.html, accessed on 15 January 2024) is a web-based pathogenicity analysis tool that leverages an SVM-based classifier to categorize variants associated with diseases [37]. PhD-SNP distinguishes between neutral and disease-associated amino acid substitutions by employing sequence and profile information. It uses machine learning algorithms trained on various sequence, structural, and functional features to predict the functional consequences of SNPs. A PhD-SNP score exceeding 0.5 signifies an amino acid substitution likely to induce disease. In our study, we utilized PhD-SNP to evaluate the pathogenicity of mutations within IGHG1.

#### 2.4.2. MutPred2

MutPred2 (http://mutpred.mutdb.org, accessed on 15 January 2024) is another web-based tool tailored to classifying amino acid substitutions as either disease-associated or neutral [36]. It aims to predict the likelihood of a given amino acid substitution being deleterious or neutral while offering insights into the potential molecular mechanisms underlying the predicted effects. MutPred2 considers various protein sequence and structure features, encompassing evolutionary conservation, the physicochemical properties of amino acids, protein domains, and structural annotations. A MutPred2 score surpassing 0.5 indicates a substitution deemed pathogenic. In our investigation, we employed MutPred2 to assess the pathogenicity of mutations within IGHG1.

### 2.5. Aggregation Propensity Analysis

Aggregation propensity analysis (of proteins) is a valuable approach to exploring how variants within a protein can influence its tendency to form aggregates [42]. SODA (http://protein.bio.unipd.it/soda/, accessed on 15 January 2024) emerged as a bioinformatics tool and web server specifically designed for predicting protein solubility based on intrinsic disorder and aggregation propensity. The tool accepts input files in either FASTA sequence or PDB structure format. SODA utilizes a combination of multiple algorithms, namely PASTA 2.0, ESpritz-NMR, and FELLS methods [43]. It ultimately provides conclusive results by comparing the solubility disparity between the wild-type and mutant proteins. In our study, we utilized SODA to assess the aggregation propensity of mutations within *IGHG1*.

### 2.6. Residual Frustration Analysis

**Figure 1:**
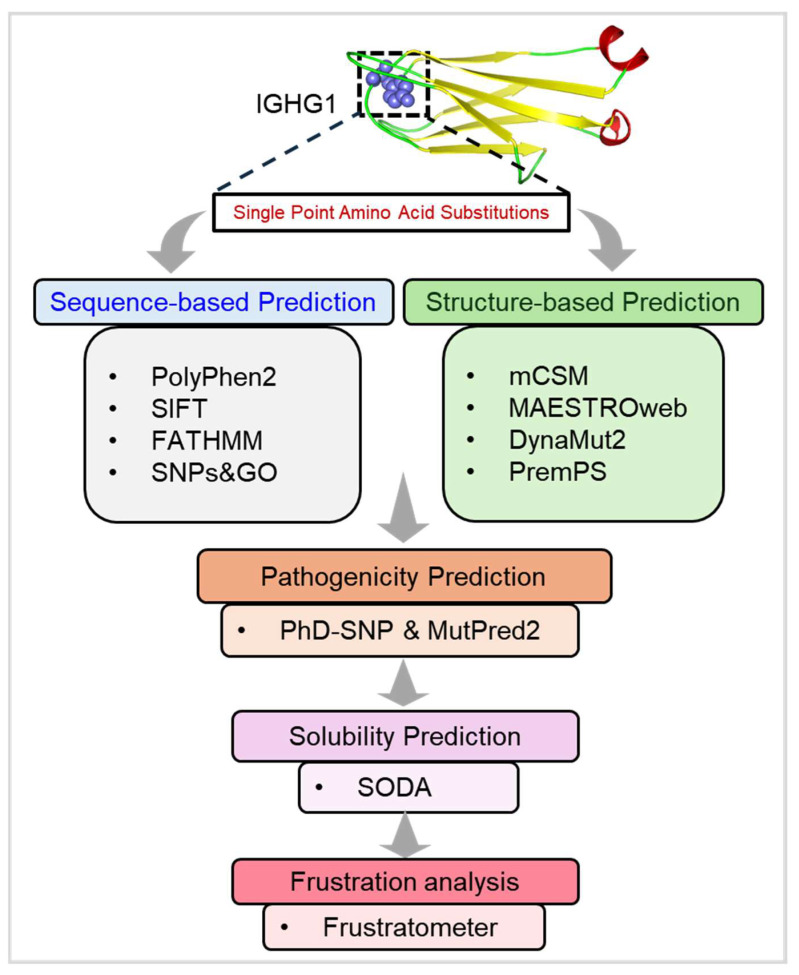
Schematic of the workflow pipeline employed in the present study. IGHG1, Immunoglobulin heavy constant gamma 1.

Residual frustration analysis in proteins provides valuable insight into the level of frustration present in the structure of a protein [44]. To explore the enduring frustration within the structure of IGHG1, we utilized the Frustratometer server (http://frustratometer.qb.fcen.uba.ar/, accessed on 15 January 2024) [45]. By using this server, we calculated individual and configurational residual indices for the structure of IGHG1. The Frustratometer evaluates the energy of a protein structure by comparing it to a set of ‘decoy’ states. Specifically, the residual frustration index between amino acids i and j is determined as a Z-score, comparing the energy of the native contact to that of N decoys. A contact is classified as highly frustrated or destabilizing if its Z-score is below 0.78. Conversely, a contact is minimally frustrated or stabilizing if the Z-score value exceeds 0.78. Contacts falling between these thresholds are considered neutral. The workflow employed in this study is depicted in Figure 1.

## 3. Results

**Figure 2:**
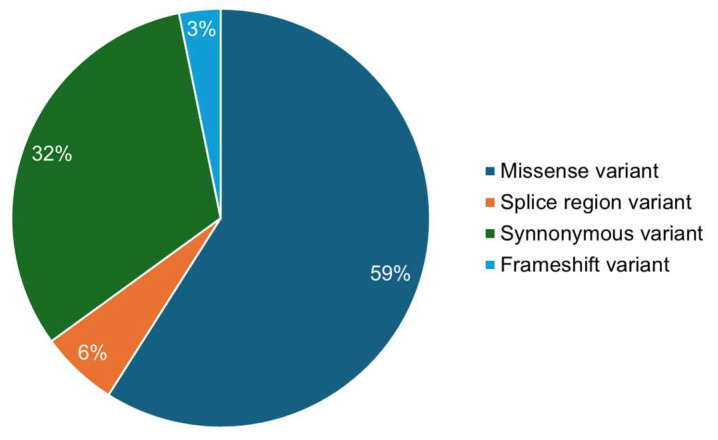
Visualization showing the single nucleotide polymorphisms (SNPs) identified within the IGHG1 gene using the dbSNP database.

A set of 126 variations in *IGHG1* sourced from the dbSNP and Ensembl databases, complemented by mutations mined from the literature (available on PubMed), was evaluated (Figure 2). Our focus centered on evaluating these substitutions’ structural and functional impacts within the CH1 region/Ig-like 1 domain of the IGHG1 protein. Our integrated approach encompassed sequence- and structure-based analyses to discover high-confidence deleterious variants. The sequence-based analysis was facilitated by four web tools: SIFT, PolyPhen2, FATHMM, and SNPs&GO. Concurrently, the structure-based approach involved utilizing mCSM, DynaMut2, MAESTROweb, and PremPS to evaluate single-point amino acid substitutions in IGHG1. Subsequently, only those mutations identified as high-confidence variants were subjected to further investigation. In order to elucidate the disease phenotypes associated with these high-confidence variants, we exploited the PhD-SNP and MutPred2 web servers, as discussed in the ensuing sections.

### 3.1. Deleterious Mutations from Sequence and Structure-Based Approaches

Integrating multiple prediction tools within the sequence-based approach minimizes false positive results and enhances the accuracy of mutation predictions [46]. Among these tools, SIFT employs protein physical properties to categorize variants as tolerated or intolerant, with a higher tolerance index indicating a lower functional impact and vice versa. Similarly, PolyPhen2 utilizes amino acid sequences to classify non-synonymous variants into the possibly damaging, probably damaging, or benign categories based on specific scores. In order to further reinforce confidence levels, the inclusion of the FATHMM and SNPs&GO tools augments the predictive capacity of the analysis. Mutations often associated with diseases have a notable impact on protein stability. Proteins can exist in either folded or unfolded states, and in thermodynamics, the Gibbs free energy between the folded (*G*f) and unfolded (*G*u) states of a protein is determined as Δ*G* = *G*u − *G*f. The alteration in protein stability and the free energy landscape is assessed by ΔΔ*G* = *G*m − *G*w, where Gm represents the mutant protein and *G*w denotes the wild-type protein. A negative ΔΔ*G* value indicates a mutation that stabilizes the protein, while a positive ΔΔ*G* value suggests variants that destabilize it.

**Figure 3:**
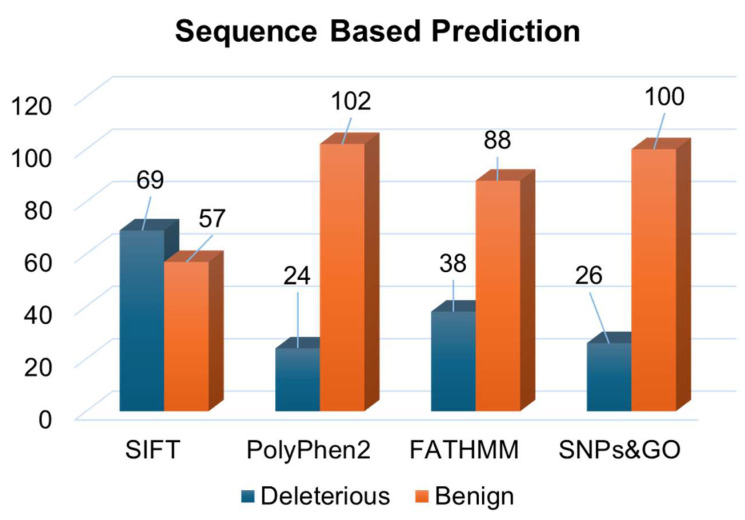
Deleterious mutations in Immunoglobulin heavy constant gamma 1predicted through sequence-based tools.

We employed four distinct structure-based prediction tools: mCSM, DynaMut2, MAESTROweb, and PremPS. These tools utilize the PDB file of the wild-type protein as input and analyze atomic co-ordinates to evaluate the stability of variants through folding free energy computation. Many of these tools employ a machine learning-centric methodology, integrating various biophysics-based techniques to forecast the influence of variants on protein stability. In the sequence-based approach, the analysis of all 126 single-point amino acid substitutions in IGHG1 yielded predictions from SIFT, PolyPhen2, FATHMM, and SNPs&GO (Supplementary Table S1). Specifically, these tools identified 69, 24, 38, and 26 substitutions as deleterious (Figure 3). This amalgamation of sequence-based prediction tools allowed us to scrutinize the potential functional impact of mutations at the molecular level.

**Figure 4:**
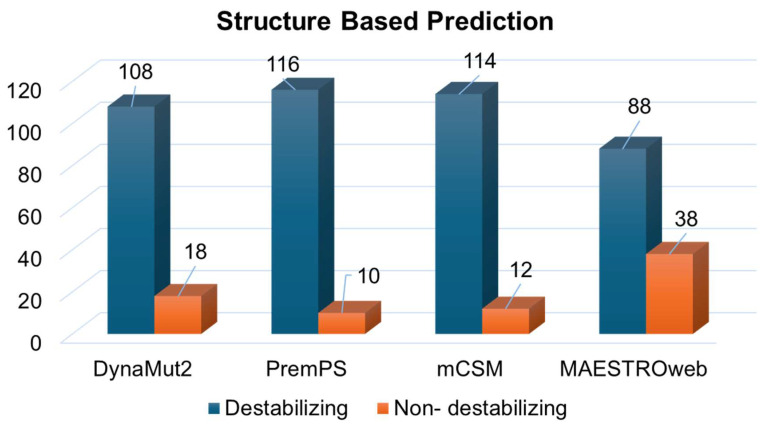
Destabilizing mutations in Immunoglobulin heavy constant gamma 1 predicted through structure-based tools.

Simultaneously, the structure-based predictions from mCSM, DynaMut2, MAESTROweb, and PremPS identified 108, 116, 114, and 88 substitutions as destabilizing mutations (Figure 4). In order to bolster confidence in our findings, only mutations predicted as deleterious by all sequence-based and structure-based tools were selected for further analysis. This filtering process resulted in 10 amino acid substitutions predicted as deleterious and destabilizing (Supplementary Table S2). Subsequently, these 10 substitutions underwent analysis to investigate their association with disease phenotypes.

### 3.2. Identification of Disease-Associated Mutations

**Figure 5:**
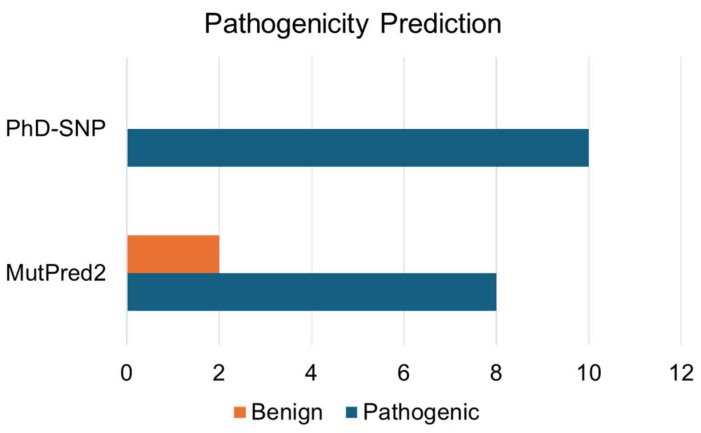
Pathogenic mutations in Immunoglobulin heavy constant gamma 1 predicted using structure-based tools.

Analyzing disease-associated protein mutations constitutes a crucial aspect of unraveling the molecular underpinnings of various complex diseases. In our investigation of disease-associated single-point mutations, we employed PhD-SNP and MutPred2. These methodologies classify mutations based on their pathogenicity scores and delineate associated disease phenotypes (Supplementary Table S3). Out of the 10 mutations analyzed, eight mutations (Y32C, Y32D, P34S, V39E, C83R, C83Y, V85M, and H87Q) were identified as pathogenic by both disease phenotype prediction tools (Figure 5). This overlapping subset of mutations signifies those with a heightened likelihood of being associated with disease phenotypes, as affirmed by the consensus prediction from both tools.

Moreover, we predicted the aggregation propensity of the protein induced by these mutations. The solubility of a protein significantly influences its functionality, as insoluble regions tend to aggregate, potentially exacerbating disease progression [42]. In order to evaluate the solubility of protein variants and discern their association with disease, we employed SODA [47]. SODA assesses various tendencies, including aggregation, disorder, helix, and strand tendencies resulting from mutations. Of the eight deleterious single-point amino acid substitutions identified via disease phenotype prediction, two substitutions (Y32C and P34S) were found to decrease the solubility of the protein (Supplementary Table S4).

### 3.3. Residual Frustration Analysis

**Figure 6:**
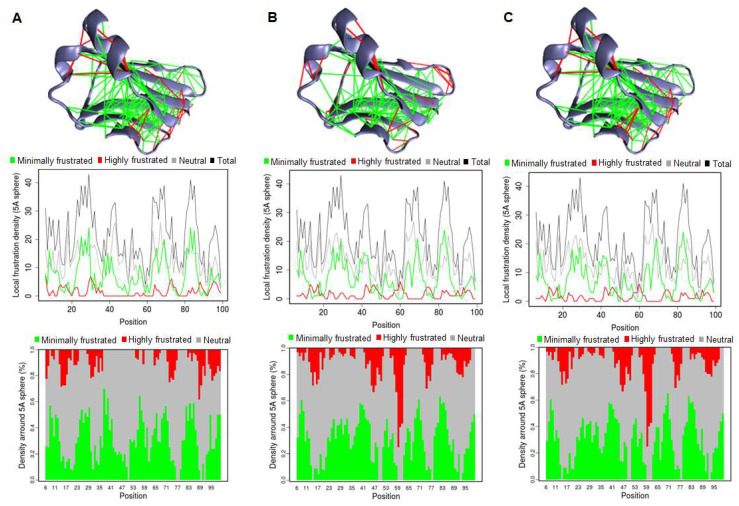
Residual frustration maps in (**A**) WT IGHG1 (**B**) Y32C and (**C**) P34S. The upper panel shows the 3D structure of Immunoglobulin heavy constant gamma 1 with a frustration index. The middle panel shows the residue–residue contact level in Immunoglobulin heavy constant gamma 1. The lower panel shows the frustration contact map in Immunoglobulin heavy constant gamma 1.

Residual frustration analysis provides invaluable insights into the intricate energy landscapes of proteins [44]. It offers a deeper understanding of the interplay between protein structure, stability, and function. By analyzing frustration within protein structures, specific locations of frustration can be identified, shedding light on critical aspects of protein behavior. Here, we conducted a structure-based analysis of local frustration within IGHG1 to discern its implications on protein stability and function (Figure 6). Frustration indices offer insights into the relative stability of native contacts compared to all potential contacts at specific locations (Figure 6A). Our analysis unveiled varying degrees of frustration within IGHG1, with the contact map showcasing significant alterations in frustration patterns in both mutants (Figure 6B,C). Particularly, the structure exhibited moderate frustration at various sites on the protein.

## 4. Discussion

HD remains a persistent challenge within the landscape of neurodegenerative disorders [48]. The molecular complexity of HD is substantial, and despite significant progress in understanding its molecular and cellular mechanisms, effective therapeutics are not yet available [49]. Studies have suggested that CSF could be a valuable source of biomarkers for HD [18,21]. IGHG1 is one of the proteins found in CSF and has been found to increase significantly in HD [22]. Considering its critical role in HD, it is reasonable to study the potential involvement of deleterious mutations in IGHG1 in the pathogenesis of this disorder. This study explored the complex landscape of deleterious mutations within the IGHG1 protein and their possible implications in HD pathogenesis.

By using a multifaceted approach that integrates sequence-based and structure-based analyses, we identified a few mutations with a high likelihood of deleterious effects on protein structure and function. By exploiting multiple computational tools and bioinformatics algorithms, initially, we recognized 10 high-confidence mutations within IGHG1, characterized by their significant impact on protein stability and their potential association with disease phenotypes. The sequence-based approach ensured the identification of mutations with the highest likelihood of playing a significant role in disease pathology.

At the same time, the structure-based predictions enhanced our confidence in identifying mutations that potentially play important roles in disease manifestation and progression. This observation underscores the potential implications of these mutations in perturbing protein solubility and highlights their relevance in disease pathogenesis [50]. Furthermore, our analysis revealed the aggregation propensity of IGHG1 variants, shedding light on their potential contribution to disease progression [51].

Developing non-dissolvable protein aggregates is a hallmark of HD [52]. Here, two substitutions (Y32C and P34S) were identified that decrease protein solubility, suggesting their potential role in protein IGHG1 and subsequent disease manifestation. Additionally, analyzing residual frustration in a protein structure is crucial in shedding light on the disease mechanism [53]. The frustration analysis provided valuable insights into the complex energy landscapes of IGHG1, highlighting regions of potential instability that could influence protein function and contribute to disease pathology. The analysis showed that mutations occurring in the minimally frustrated residues of IGHG1 could disrupt stability, thereby affecting the function of the protein and potentially contributing to HD pathogenesis. This analysis underscores the importance of considering local frustration in elucidating the molecular mechanisms underlying disease pathology.

## 5. Conclusions

Our findings highlight the importance of computational mutational analysis in unraveling the molecular basis of complex diseases such as HD. This study contributes to the growing body of knowledge on understanding the pathogenesis of HD and lays the groundwork for future research endeavors to develop targeted therapeutic interventions. Ultimately, the study emphasizes the significance of advanced computational approaches to gain deeper insights into the molecular mechanisms driving disease pathology.

## Data Availability

The original contributions presented in the study are included in the article/Supplementary Materials; further inquiries can be directed to the corresponding author.

## Supplementary Materials

The following supporting information can be downloaded at https://www.mdpi.com/article/10.3390/jpm14040380/s1, Table S1: Prediction of deleterious mutations in IGHG1 using sequence-based tools; Table S2: Prediction of destabilizing mutations in IGHG1 using structure-based tools; Table S3: Prediction of pathogenic mutations in IGHG1 using structure-based tools; Table S4: Prediction of aggregation propensity of mutations in IGHG1.
