# Supplementary material for: Elucidating the Impact of Deleterious Mutations on IGHG1 and Their Association with Huntington’s Disease
Source: J Pers Med. 2024 Apr 1;14(4):380. doi: 10.3390/jpm14040380 (PMC11050829; doi:10.3390/jpm14040380)
Supplement: Supplementary file 1 [file jpm-14-00380-s001.zip › jpm-2939778-supplementary.pdf]

Table S1: Prediction of deleterious mutations in IGHG1 using sequence-based tools.

S. No.	Mutation	SIFT Class	PolyPhen Class	FATHMM	SNPs&GO
1.	A12P	Deleterious	Benign	Tolerated	Neutral
2.	A12T	Tolerated	Benign	Tolerated	Neutral
3.	A12V	Deleterious	Benign	Tolerated	Neutral
4.	A23E	Deleterious	Benign	Damaging	Disease
5.	A23V	Tolerated	Benign	Tolerated	Neutral
6.	A24S	Tolerated	Benign	Tolerated	Neutral
7.	A24T	Tolerated	Benign	Tolerated	Neutral
8.	A45D	Deleterious	Benign	Tolerated	Disease
9.	A45S	Tolerated	Benign	Tolerated	Neutral
10.	A45T	Tolerated	Benign	Tolerated	Neutral
11.	A55P	Deleterious	Benign	Tolerated	Neutral
12.	A55T	Tolerated	Benign	Tolerated	Neutral
13.	C83R	Deleterious	Probably Damaging	Damaging	Disease
14.	C83Y	Deleterious	Probably Damaging	Damaging	Disease
15.	D95N	Tolerated	Benign	Tolerated	Neutral
16.	E35K	Deleterious	Benign	Tolerated	Neutral
17.	E99G	Tolerated	Benign	Tolerated	Neutral
18.	E99V	Tolerated	Benign	Tolerated	Neutral
19.	F53C	Deleterious	Possibly Damaging	Damaging	Disease
20.	F53L	Deleterious	Benign	Tolerated	Neutral
21.	F53Y	Tolerated	Benign	Tolerated	Neutral
22.	G20E	Tolerated	Benign	Tolerated	Neutral
23.	G20R	Tolerated	Benign	Tolerated	Neutral
24.	G20W	Deleterious	Benign	Damaging	Neutral
25.	G21D	Tolerated	Benign	Tolerated	Neutral
26.	G21S	Tolerated	Benign	Tolerated	Neutral
27.	G21V	Deleterious	Benign	Damaging	Neutral
28.	G26D	Deleterious	Benign	Damaging	Disease
29.	G49D	Deleterious	Benign	Tolerated	Disease
30.	G49S	Tolerated	Benign	Tolerated	Neutral
31.	G49V	Deleterious	Benign	Tolerated	Disease
32.	G61V	Deleterious	Possibly Damaging	Damaging	Neutral
33.	G77D	Tolerated	Benign	Tolerated	Neutral
34.	G77S	Tolerated	Benign	Tolerated	Neutral
35.	H87Q	Deleterious	Probably Damaging	Damaging	Disease
36.	I82T	Tolerated	Benign	Tolerated	Neutral
37.	K16E	Tolerated	Benign	Tolerated	Neutral
38.	K16Q	Tolerated	Benign	Tolerated	Neutral
39.	K16R	Tolerated	Benign	Tolerated	Neutral
40.	K88Q	Tolerated	Benign	Tolerated	Neutral

41.	K88R	Deleterious	Benign	Tolerated	Neutral
42.	K93N	Tolerated	Benign	Tolerated	Neutral
43.	K96R	Deleterious	Benign	Tolerated	Neutral
44.	K97E	Tolerated	Benign	Tolerated	Neutral
45.	K97I	Tolerated	Possibly Damaging	Damaging	Neutral
46.	K97R	Tolerated	Benign	Tolerated	Neutral
47.	K97T	Tolerated	Benign	Tolerated	Neutral
48.	L57P	Deleterious	Benign	Damaging	Disease
49.	L57V	Deleterious	Benign	Tolerated	Neutral
50.	L62F	Tolerated	Benign	Tolerated	Neutral
51.	L62H	Deleterious	Probably Damaging	Tolerated	Neutral
52.	L62P	Deleterious	Benign	Tolerated	Neutral
53.	L65F	Deleterious	Benign	Tolerated	Neutral
54.	L76F	Deleterious	Benign	Tolerated	Neutral
55.	N84K	Tolerated	Benign	Tolerated	Neutral
56.	N84S	Tolerated	Benign	Tolerated	Neutral
57.	N86D	Tolerated	Benign	Tolerated	Neutral
58.	N86T	Tolerated	Benign	Tolerated	Neutral
59.	N91I	Deleterious	Benign	Damaging	Neutral
60.	P10H	Deleterious	Possibly Damaging	Damaging	Neutral
61.	P10S	Deleterious	Benign	Damaging	Neutral
62.	P13A	Deleterious	Benign	Tolerated	Neutral
63.	P13S	Tolerated	Benign	Tolerated	Neutral
64.	P13T	Deleterious	Benign	Tolerated	Neutral
65.	P34S	Deleterious	Probably Damaging	Damaging	Disease
66.	P36L	Deleterious	Benign	Tolerated	Disease
67.	P54L	Deleterious	Benign	Damaging	Disease
68.	P72L	Deleterious	Benign	Damaging	Neutral
69.	P72R	Deleterious	Possibly Damaging	Tolerated	Neutral
70.	P72S	Tolerated	Benign	Tolerated	Neutral
71.	P72T	Tolerated	Benign	Tolerated	Neutral
72.	P89S	Deleterious	Benign	Tolerated	Neutral
73.	Q58P	Deleterious	Benign	Tolerated	Disease
74.	Q79H	Deleterious	Benign	Damaging	Neutral
75.	Q79K	Tolerated	Benign	Tolerated	Neutral
76.	S14C	Tolerated	Benign	Tolerated	Neutral
77.	S17T	Tolerated	Benign	Tolerated	Neutral
78.	S19Y	Deleterious	Benign	Damaging	Neutral
79.	S40L	Deleterious	Benign	Tolerated	Neutral
80.	S43T	Tolerated	Benign	Tolerated	Neutral
81.	S48G	Tolerated	Benign	Tolerated	Disease
82.	S48N	Deleterious	Benign	Tolerated	Disease

83.	S48R	Tolerated	Benign	Tolerated	Disease
84.	S59F	Deleterious	Probably Damaging	Damaging	Disease
85.	S59Y	Deleterious	Probably Damaging	Damaging	Disease
86.	S60A	Deleterious	Possibly Damaging	Tolerated	Neutral
87.	S64C	Deleterious	Benign	Damaging	Neutral
88.	S67R	Deleterious	Probably Damaging	Damaging	Neutral
89.	S75N	Tolerated	Benign	Tolerated	Neutral
90.	S75R	Tolerated	Benign	Tolerated	Neutral
91.	S75T	Tolerated	Benign	Tolerated	Neutral
92.	S7L	Deleterious	Benign	Tolerated	Neutral
93.	S90N	Deleterious	Benign	Tolerated	Neutral
94.	S90R	Deleterious	Possibly Damaging	Tolerated	Neutral
95.	T18N	Deleterious	Benign	Tolerated	Neutral
96.	T22K	Tolerated	Benign	Tolerated	Neutral
97.	T38A	Deleterious	Benign	Tolerated	Neutral
98.	T38M	Deleterious	Benign	Damaging	Disease
99.	T47I	Deleterious	Benign	Damaging	Disease
100.	T47N	Tolerated	Benign	Tolerated	Neutral
101.	T47S	Tolerated	Benign	Tolerated	Neutral
102.	T52I	Deleterious	Benign	Tolerated	Neutral
103.	T52N	Tolerated	Benign	Tolerated	Neutral
104.	T70I	Tolerated	Benign	Damaging	Neutral
105.	T80I	Deleterious	Benign	Tolerated	Neutral
106.	T92I	Deleterious	Benign	Tolerated	Neutral
107.	V37A	Deleterious	Probably Damaging	Damaging	Disease
108.	V37M	Deleterious	Benign	Damaging	Neutral
109.	V39E	Deleterious	Probably Damaging	Damaging	Disease
110.	V39M	Tolerated	Benign	Damaging	Neutral
111.	V50A	Deleterious	Benign	Tolerated	Neutral
112.	V50L	Tolerated	Benign	Tolerated	Neutral
113.	V50M	Deleterious	Benign	Tolerated	Neutral
114.	V56A	Tolerated	Benign	Tolerated	Neutral
115.	V68L	Tolerated	Benign	Tolerated	Neutral
116.	V68M	Tolerated	Benign	Tolerated	Neutral
117.	V71G	Deleterious	Probably Damaging	Damaging	Neutral
118.	V71L	Tolerated	Benign	Tolerated	Neutral
119.	V71M	Deleterious	Possibly Damaging	Damaging	Neutral
120.	V85M	Deleterious	Possibly Damaging	Damaging	Disease
121.	V94G	Deleterious	Probably Damaging	Damaging	Neutral
122.	V94M	Deleterious	Benign	Damaging	Neutral
123.	V98L	Tolerated	Benign	Tolerated	Neutral
124.	Y32C	Deleterious	Probably Damaging	Damaging	Disease

125.	Y32D	Deleterious	Probably Damaging	Damaging	Disease
126.	Y81C	Deleterious	Probably Damaging	Damaging	Neutral

Table S2: Prediction of destabilizing mutations in IGHG1 using structure-based tools.

S. No	Mutation	mCSM	DynaMut2	MAESTROweb	PremPS
1.	A12P	−0.27	0.5	Destabilizing	0.537
2.	A12T	−0.37	0.33	Destabilizing	0.246
3.	A12V	−0.7	−0.2	Destabilizing	−0.401
4.	A23E	−1.08	1.17	Destabilizing	2.379
5.	A23V	−1.03	−0.24	Destabilizing	0.256
6.	A24S	−1.02	0.4	Destabilizing	0.422
7.	A24T	−1.1	0.23	Destabilizing	−0.196
8.	A45D	−0.31	0.55	Destabilizing	0.146
9.	A45S	0.18	0.14	Destabilizing	0.611
10.	A45T	0.15	0.27	Destabilizing	0.215
11.	A55P	−0.35	0.4	Destabilizing	0.799
12.	A55T	−1.05	0.35	Destabilizing	0.105
13.	C83R	−1.13	2.26	Destabilizing	3.141
14.	C83Y	−1.34	0.94	Destabilizing	1.436
15.	D95N	−1.37	0.48	Destabilizing	0.988
16.	E35K	−0.51	0.25	Destabilizing	−0.398
17.	E99G	0.19	0.24	Destabilizing	−0.465
18.	E99V	0.2	−0.12	Stabilizing	−0.536
19.	F53C	0.96	0.4	Destabilizing	0.229
20.	F53L	0.65	0.05	Destabilizing	0.125
21.	F53Y	−0.03	0.08	Destabilizing	−0.119
22.	G20E	−0.15	0.58	Destabilizing	0.005
23.	G20R	−0.11	0.69	Destabilizing	0.059
24.	G20W	−1.57	0.4	Destabilizing	0.782
25.	G21D	−0.4	0.65	Destabilizing	0.992
26.	G21S	−0.21	0.31	Destabilizing	0.757
27.	G21V	−1.39	0.36	Destabilizing	1.204
28.	G26D	−1.72	1.25	Destabilizing	1.553
29.	G49D	−0.57	0.57	Destabilizing	0.551
30.	G49S	−0.41	0.44	Destabilizing	0.067
31.	G49V	−2.4	0.26	Destabilizing	0.982
32.	G61V	−1.21	0.33	Destabilizing	0.756
33.	G77D	−0.56	0.51	Destabilizing	−0.223
34.	G77S	−0.49	0.33	Destabilizing	0.089
35.	H87Q	−1.25	1.01	Destabilizing	2.19
36.	I82T	−2.54	1.07	Highly Destabilizing	1.713
37.	K16E	−0.33	0.06	Destabilizing	−0.453

38.	K16Q	−0.15	0.12	Stabilizing	−0.395
39.	K16R	−0.08	0.2	Destabilizing	−0.302
40.	K88Q	−0.08	0.27	Stabilizing	−0.536
41.	K88R	−0.37	0.37	Destabilizing	−0.657
42.	K93N	−0.31	0.81	Destabilizing	−0.24
43.	K96R	−0.42	0.37	Destabilizing	−0.346
44.	K97E	0.04	0.45	Stabilizing	0.2
45.	K97I	1.27	−0.12	Stabilizing	−1.06
46.	K97R	−0.72	−0.02	Destabilizing	−1
47.	K97T	−0.2	0.28	Destabilizing	−0.523
48.	L57P	−0.11	0.65	Destabilizing	0.151
49.	L57V	0.12	0.2	Destabilizing	0.54
50.	L62F	−0.76	0.19	Destabilizing	−0.175
51.	L62H	−0.25	0.92	Destabilizing	−0.159
52.	L62P	0.46	0.91	Destabilizing	0.176
53.	L65F	−1.51	0.67	Destabilizing	1.273
54.	L76F	−0.66	0.12	Destabilizing	0.044
55.	N84K	−0.64	0.94	Destabilizing	0.847
56.	N84S	−0.98	0.99	Destabilizing	0.804
57.	N86D	−1.25	0.54	Destabilizing	0.099
58.	N86T	−0.27	0.35	Destabilizing	0.483
59.	N91I	−0.53	−0.08	Destabilizing	0.138
60.	P10H	−1.72	0.94	Destabilizing	0.943
61.	P10S	−2.03	0.81	Destabilizing	0.943
62.	P13A	−0.23	0.42	Destabilizing	0.245
63.	P13S	−0.25	0.57	Destabilizing	0.766
64.	P13T	−0.18	0.51	Destabilizing	0.177
65.	P34S	−2.26	0.8	Highly Destabilizing	1.078
66.	P36L	−0.93	0.16	Destabilizing	−0.045
67.	P54L	−0.63	0.12	Destabilizing	−0.195
68.	P72L	−0.31	0.13	Destabilizing	0.241
69.	P72R	0.35	0.7	Stabilizing	−0.149
70.	P72S	−0.27	0.64	Destabilizing	0.212
71.	P72T	0.05	0.47	Destabilizing	−0.061
72.	P89S	−0.32	0.5	Destabilizing	0.167
73.	Q58P	0.02	0.13	Stabilizing	−0.297
74.	Q79H	−0.31	0.01	Destabilizing	−0.175
75.	Q79K	0.26	0.11	Stabilizing	−0.684
76.	S14C	−0.42	−0.31	Destabilizing	1.141
77.	S17T	−0.11	0.05	Destabilizing	−0.049
78.	S19Y	−1.8	0.05	Destabilizing	0.269
79.	S40L	−0.77	0.12	Destabilizing	−0.053

80.	S43T	−0.78	0.14	Destabilizing	0.112
81.	S48G	−1.25	0.44	Destabilizing	1.059
82.	S48N	−0.53	0.3	Destabilizing	0.413
83.	S48R	−0.83	0.41	Destabilizing	0.924
84.	S59F	−1.07	0	Destabilizing	0.084
85.	S59Y	−1.1	0.02	Destabilizing	0.057
86.	S60A	−0.24	0.3	Destabilizing	−0.094
87.	S64C	−0.46	0.59	Destabilizing	0.15
88.	S67R	−0.52	1.11	Destabilizing	1.445
89.	S75N	−0.2	0.25	Destabilizing	0.297
90.	S75R	−0.48	0.36	Destabilizing	0.119
91.	S75T	−0.27	−0.1	Destabilizing	−0.262
92.	S7L	0.24	−0.32	Stabilizing	−0.131
93.	S90N	−1.5	0.63	Destabilizing	0.061
94.	S90R	−1.13	0.71	Destabilizing	0.313
95.	T18N	0.6	0.68	Stabilizing	0.324
96.	T22K	−0.4	0.59	Destabilizing	0.663
97.	T38A	−0.69	0.55	Destabilizing	0.686
98.	T38M	0.23	0.23	Stabilizing	0.044
99.	T47I	−0.68	0.06	Destabilizing	0.16
100.	T47N	0.02	0.22	Stabilizing	−0.651
101.	T47S	−0.31	0.17	Destabilizing	−0.31
102.	T52I	−0.66	0.08	Destabilizing	−0.434
103.	T52N	−1.53	0.48	Destabilizing	0.778
104.	T70I	−0.52	0.12	Destabilizing	−0.414
105.	T80I	−0.73	0.08	Destabilizing	0.533
106.	T92I	−0.79	0.18	Destabilizing	0.176
107.	V37A	−2.38	1.27	Destabilizing	1.668
108.	V37M	−0.79	0.71	Destabilizing	0.792
109.	V39E	−0.8	1.65	Destabilizing	2.844
110.	V39M	−0.82	0.72	Destabilizing	0.079
111.	V50A	−0.28	0.19	Destabilizing	−0.25
112.	V50L	−0.5	0.06	Destabilizing	−0.549
113.	V50M	−0.53	0.06	Destabilizing	−0.818
114.	V56A	−1.38	0.69	Destabilizing	0.648
115.	V68L	−0.84	0.41	Destabilizing	0.093
116.	V68M	−1.17	0.29	Destabilizing	0.539
117.	V71G	−1.16	1.53	Destabilizing	2.1
118.	V71L	−0.65	0.5	Destabilizing	0.197
119.	V71M	−0.45	0.65	Destabilizing	0.336
120.	V85M	−1.93	1.19	Destabilizing	1.534
121.	V94G	−2.99	1.92	Destabilizing	1.681

122.	V94M	−0.75	0.6	Destabilizing	0.113
123.	V98L	−0.45	0.25	Destabilizing	0.563
124.	Y32C	−2.26	2.48	Destabilizing	1.746
125.	Y32D	−4.21	2.44	Highly Destabilizing	4.089
126.	Y81C	−0.53	1.18	Destabilizing	0.8

Table S3: Prediction of pathogenic mutations in IGHG1 using structure-based tools.

S. No.	Mutation	PhD-SNP	MutPred2	MutPred2 remarks
1.	C83R	Disease	0.904	Loss of Disulfide linkage at C83 (Pr = 0.45 | P = 4.3e-04); Altered Metal binding (Pr = 0.31 | P = 0.01); Gain of Intrinsic disorder (Pr = 0.31 | P = 0.04); Altered Ordered interface (Pr = 0.29 | P = 0.03); Altered Transmembrane protein (Pr = 0.27 | P = 8.4e-04); Altered Stability (Pr = 0.22 | P = 0.01)
2.	C83Y	Disease	0.866	Loss of Disulfide linkage at C83 (Pr = 0.45 | P = 4.3e-04); Altered Disordered interface (Pr = 0.31 | P = 0.02); Altered Metal binding (Pr = 0.30 | P = 0.01); Altered Transmembrane protein (Pr = 0.28 | P = 4.6e-04); Altered Ordered interface (Pr = 0.28 | P = 0.05); Gain of Loop (Pr = 0.26 | P = 0.04); Altered Stability (Pr = 0.19 | P = 0.01)
3.	H87Q	Disease	0.713	Altered Metal binding (Pr = 0.39 | P = 6.9e-03); Gain of Intrinsic disorder (Pr = 0.32 | P = 0.03); Loss of Disulfide linkage at C83 (Pr = 0.25 | P = 5.8e-03); Altered Transmembrane protein (Pr = 0.24 | P = 2.1e-03); Gain of GPI-anchor amidation at N91 (Pr = 0.02 | P = 0.01)
4.	P34S	Disease	0.552	Altered Transmembrane protein (Pr = 0.29 | P = 4.0e-04); Gain of Sulfation at Y32 (Pr = 0.01 | P = 0.04)
5.	S59Y	Disease	0.428	-
6.	V37A	Disease	0.273	-
7.	V39E	Disease	0.877	Altered Transmembrane protein (Pr = 0.33 | P = 4.9e-05); Loss of Strand (Pr = 0.27 | P = 0.02)
8.	V85M	Disease	0.6	Altered Metal binding (Pr = 0.28 | P = 0.02); Gain of Disulfide linkage at C83 (Pr = 0.26 | P = 4.6e-03); Altered Transmembrane protein (Pr = 0.23 | P = 2.2e-03)
9.	Y32C	Disease	0.687	Altered Transmembrane protein (Pr = 0.56 | P = 0.0e+00); Gain of Disulfide linkage at C27 (Pr = 0.26 | P = 4.3e-03); Loss of Sulfation at Y32 (Pr = 0.01 | P = 0.04)
10.	Y32D	Disease	0.797	Altered Transmembrane protein (Pr = 0.31 | P = 1.2e-04); Loss of Disulfide linkage at C27 (Pr = 0.21 | P = 0.02); Loss of Sulfation at Y32 (Pr = 0.01 | P = 0.04)

Table S4: Prediction of aggregation propensity of mutations in IGHG1.

S. No.	Mutations	Helix	Strand	Aggregation	Disorder	SODA	Solubility
1.	Wild type	0.217	0.32	−8.192	0.052		
2.	Y32C	−0.65	0.389	−0.143	−0.02	−1.182	Less soluble
3.	Y32D	−3.223	2.292	8.089	0.627	7.756	More soluble
4.	P34S	0.694	−0.101	−9.467	0.045	−8.294	Less soluble
5.	V39E	0.758	−2.479	15.097	−0.075	12.471	More soluble
6.	C83R	1.189	−1.682	13.423	0.544	14.401	More soluble
7.	C83Y	−0.043	0.173	−0.467	0.033	0.337	More soluble
8.	V85M	0.491	−0.962	11.153	−0.021	10.455	More soluble
9.	H87Q	0.028	−0.106	0.519	0.207	0.814	More soluble
